# A high-throughput lipidomics and transcriptomic approach reveals novel compounds from sugarcane linked with promising therapeutic potential against COVID-19

**DOI:** 10.3389/fnut.2022.988249

**Published:** 2022-09-02

**Authors:** Muhammad Junaid Rao, Muhammad Tahir ul Qamar, Dongxin Wang, Qurban Ali, Li Ma, Shijian Han, Mingzheng Duan, Lihua Hu, Lingqiang Wang

**Affiliations:** ^1^State Key Laboratory for Conservation and Utilization of Subtropical Agro-Bioresources, Guangxi Key Laboratory of Sugarcane Biology, College of Agriculture, Guangxi University, Nanning, China; ^2^College of Life Science and Technology, Guangxi University, Nanning, China; ^3^Key Laboratory of Integrated Management of Crop Diseases and Pests, Department of Plant Pathology, College of Plant Protection, Nanjing Agricultural University, Ministry of Education, Nanjing, China

**Keywords:** sugarcane rind, metabolomics approach, lipidomics, transcriptomic, pharmacological value, COVID-19

## Abstract

Sugarcane (*Saccharum* ssp., Poaceae) provides enormous metabolites such as sugars, lipid, and other dietary metabolites to humans. Among them, lipids are important metabolites that perform various functions and have promising pharmacological value. However, in sugarcane, few studies are focusing on lipidomics and few lipid compounds were reported, and their pharmacological values are not explored yet. The transcriptomic and widely targeted lipidomics approach quantified 134 lipid compounds from the rind of six sugarcane genotypes. These lipid compounds include 57 fatty acids, 30 lysophosphatidylcholines, 23 glycerol esters, 21 lysophosphatidylethanolamines, 2 phosphatidylcholines, and 1 sphingolipid. Among them, 119 compounds were first time reported in sugarcane rind. Seventeen lipids compounds including 12 fatty acids, 2 glycerol lipids, LysoPC 16:0, LysoPE 16:0, and choline alfoscerate were abundantly found in the rind of sugarcane genotypes. From metabolic and transcriptomic results, we have developed a comprehensive lipid metabolic pathway and highlighted key genes that are differentially expressed in sugarcane. Several genes associated with α-linolenic acid and linoleic acid biosynthesis pathways were highly expressed in the rind of the ROC22 genotype. ROC22 has a high level of α-linolenic acid (an essential fatty acid) followed by ROC16. Moreover, we have explored pharmacological values of lipid compounds and found that the 2-linoleoylglycerol and gingerglycolipid C have strong binding interactions with 3CL^pro^ of SARS-CoV-2 (severe acute respiratory syndrome coronavirus 2) and these compounds can be utilized against SARS-CoV-2 as therapeutic agents. The transcriptome, metabolome, and bioinformatics analysis suggests that the sugarcane cultivars have a diversity of lipid compounds having promising therapeutic potential, and exploring the lipid metabolism will help to know more compounds that have promising cosmetic and pharmacological value.

## Introduction

Sugarcane crop contributes to 80% of world sugar production and major feedstock for bioenergy production. Sugarcane belongs to the perennial grass family “Poaceae,” which is the fifth major cultivated crop in the world (http://faostat.fao.org/) (1). Brazil, India, and China are leading sugarcane-producing countries (2, 3). Sugarcane residues are also utilized as fodder, organic fertilizer, extraction of several essential color components, and several byproducts, and also burned for electricity generation (4). In addition, sugarcane contains health-promoting lipid compounds such as linoleic acid and α-linolenic acid also known as omega-6 fatty acid and omega-3 essential fatty acids, respectively (5). Some promising biologically active compounds such as policosanol that have unique nutritive and pharmacological values have been isolated from sugarcane (3, 6); recently, various phenolic compounds including phenolic acids (such as p-ferulic acid and coumaric acid) have been extracted from sugarcane rind which executes weight loss effect in rats (7, 8). Sugarcane rind is a storehouse of several health-promoting metabolites that have significant nutritive, cosmetic, and pharmacological values (7, 9). It is revealed that sugarcane rind possesses enormous kinds of metabolites such as lipids, carbohydrates, phenolics, alkaloids, aromatic amino acids, anthocyanins, phytosterols, flavonoids, triterpenoids, carotenoids, and other phytocompounds (10–12), which have diverse health-promoting properties such as anticancer, antiplatelet, antioxidant activity, antifungal, antibacterial, antiviral, anti-HIV activities quenching free radicals, and anti-inflammatory activities (13, 14).

Lipids are small amphiphilic or hydrophobic molecules that play important roles in signaling, structural component, and energy storage. Lipids include waxes, sterols, phospholipids, and fatty acids and they are highly soluble in an organic solvent but insoluble in water (14, 15). The various combination of isoprene and ketoacyl building blocks arise huge structural complexities that make lipids classification often challenging (15). The lipids are classified into the sub-categories such as free fatty acids, sphingolipids, glycerophospholipids, and glycerolipids (15, 16). The glycerophospholipids are further divided into phosphatidylcholine (PC) which is further converted to phosphatidylethanolamine (PE) (17). The lysophosphatidylcholines (LPCs) are derived from PC whereas the lysophosphatidylethanolamines (LPEs) are derived from PE. The LPCs and LPEs are found in the cell membrane of several plant species as minor phospholipids. The fatty acids compounds such as myristic acid, stearic acid, lauric acid, and palmitic acid are famous phytocompounds that significantly inhibit microbial activity (18, 19). Glycerophosphocholine (choline alfoscerate) is commonly utilized against various human neurodegenerative disorders such as Parkinson's disease and Alzheimer's disease (20). Besides this, the fat compounds are also combined with proteins known as lipoproteins. Moreover, lipids also act as light-absorbing pigments, enzyme cofactors, hydrophobic proteins, and electron carriers (14). Lipid compounds are widely utilized as a nutritive component, in food, and cosmetic industries and recently in nanotechnology (2, 21). The complex structures and identification of new lipid compounds will continue to be an imperious research field that opens new doors to explore plant lipidomics.

In sugarcane rind, only a few lipid compounds have been reported before. Previous studies mostly focused on the production of sugar and biofuel, whereas the diversity of lipid compounds and their pharmacological value is less explored. Sugarcane is the fifth largest cultivated crop in the world, thus its rind is widely available for extracting different valuable compounds that have potential pharmacological and cosmetic value. In this study, we used a transcriptomic and widely targeted liquid chromatography-mass with a tandem spectrometry-mass (LC-MS/MS) approach to explore the biosynthesis pathway and diversity of lipids in sugarcane genotypes. Additionally, we identify the major lipid compounds in the sugarcane and highlighted their distinctive pharmacological value which will be valuable for future lipidomics research.

## Materials and methods

The rind samples were harvested from six cultivated sugarcane genotypes including ROC22 (ROC22), Yuetang93/159 (YT93/159), Taitang 172 (F172), Taitang134 (F134), ROC16 (ROC16), and Yuetang71/210 (YT71/210) which were planted in Guangxi University (experimental area), China. From each variety, rind samples were harvested (having three biological repeats) at 6 months stage (January 2021) and frozen immediately for transcriptome and LC-MS/MS analysis.

### Transcriptomic analysis

TRIzol reagent procedure was adopted for total RNA extraction from sugarcane rind, referring to the company's guidelines (Invitrogen), followed by removal of genomic DNA by DNase I (Takara). From total RNA, library construction was achieved by the TruSeq RNA kit, and sequencing was performed according to the producer's procedures (Illumina, San Diego, CA). For sequencing, Illumina HiSeq 2500 was used for the production of a (paired-end) RNA-seq sequencing library. Annotation of unigenes was accomplished by using publicly available protein databases such as Pfam (protein family), Gene Ontology, and KEGG. GOseq R package was performed for enrichment analysis (gene ontology) of differentially expressed genes (DEGs), in which gene bias length was adjusted (22, 23). *Saccharum spontaneum* genome was used as a reference genome (publically available Saccharum Genome Database website; http://sugarcane.zhangjisenlab.cn/sgd/html/download.html). Gene ontology (GO) terms having <0.05 *P*-value were measured as significantly enriched by DEGs. For statistical enrichment of DEGs in KEGG (http://www.genome.jp/kegg/) pathways, KOBAS software was used. The transcriptomic data is submitted online and have PRJNA824938 accession number. The raw transcriptomic data of thirty-five genes accompanying lipid metabolism is represented in Supplementary File 1.

### Chemical reagents for metabolic analysis and rind samples preparation and extraction

The MilliQ (Bradford, USA) water was used in all metabolic analysis processes. Hydrochloric acid and formic acid were obtained from Xinyang Chemical Reagent (Hunan, China) and Sigma-Aldrich (St. Louis, MO, USA), respectively. An internal standard from Sigma-Aldrich was purchased for quality control (http://www.sigmaaldrich.com/united-states.html) whereas the reagents methanol (MeOH, grade-HPLC) and acetonitrile were purchased from Merck (Darmstadt, Germany). The 1 mg/mL in 50% MeOH concentration of stock solutions were prepared for standards and stored at−20°C. The working solutions were made before analysis by dissolving the stock solutions with 50% MeOH.

For lipids quantitative analysis, multiple reactions monitoring (MRM) (http://en.metware.cn/) analysis was managed by Metware Co., Ltd., (Wuhan, China). The zirconia bead was used to crush (mixer mill, MM 400, Retsch) the rind freeze-dried samples at 30 Hz for 1.5 min. The 50 mg sugarcane rind was extracted with 1 mL methanol. After that, the extract was ultrasound and vortex for 5 min each followed by centrifuge at 4°C for 3 min at 12,000 × g and this step was repeated to extract the rind residue for a second time. Before LC-MS/MS analysis, the supernatants were together and filtrated (0.22 μm, Anpel).

### Chromatography and instrument conditions and metabolic analysis

Ultra-performance liquid chromatography (UPLC) was used for sample extracts analysis combined with electrospray ionization tandem mass spectrometry (ESI-MS/MS) system (UPLC-ESI-MS/MS) (Triple Quadrupole Applied Biosystems AB4500, SCIEX brand, China; https://sciex.com.cn/) (24). The analytical conditions were as follows, methanol (0.1% formic acid); solvent system, water (0.1% formic acid): UPLC: column C18 (1.7 μm, 2.1 mm^*^100 mm); gradient program, 0 min 95:5V/V, 6 min 50:50 V/V, 12 min 5:95 V/V, wait 2 min, 14 min 95:5 V/V; wait 2 min; injection volume: 2 μL; temperature, 40°C; flow rate, 0.35 mL/min. On the other hand, the effluent was linked to electrospray ionization (ESI) triple quadrupole-linear ion trap (QTRAP)-MS.

ESI source operation parameters include temperature 550°C; turbo spray, ion source; ion spray 5,500 voltage (V) and negative 4,500 V mode; curtain (CUR) gas, ion source gas (GSI), and gas (GSII), and were set at 25, 50, and 60 psi, consistently. The MRM individual transitions were developed to further optimize the de-clustering potential and collision energy. According to the elution of metabolites, monitoring of MRM transition was specifically set for each period. The triple quadrupole (QQQ) and linear ion trap (LIT) were accomplished by QTRAP, equipped with an ESI Ion-Spray Turbo interface, operating in positive and negative ion mode, governed by Analyst 1.6.3 (AB Sciex, China) software.

For MRM metabolic analysis firstly, we eliminated the interference of the target (precursor ion) substance by screening, then the molecular weight of the parallel ion is omitted. Then, precursor ions were persuaded to ionize in the collision chamber to form several fragment ions (Supplementary Figure 1). Subsequently, to make the quantification more reliable, the fragment ions were filtered by using QQQ, to attain the specific or required (removing non-target ion interference) fragment ion. The individual metabolite peak area and mass spectrum peaks were integrated for all compounds and then matched in different samples (25). The retention time (RT) and secondary spectrum fragment of all samples were matched (one by one) with the RT and secondary spectra of the Metware (http://en.metware.cn/) database. For more precise widely targeted metabolomics results, a low-resolution mass spectrometer with higher sensitivity was used, and the lipids were characterized as RT, MS2 (secondary spectrum), Q1, and Q3).

### Lipids pharmacological value assessment

The lipid compounds were explored for their pharmacological potential through docking against SARS-CoV-2. For molecular docking analyses, the same protocol was utilized as published in our previous study (26). A molecular operating environment (MOE) tool was used for this analysis (27). Briefly, the 3D structures of all lipid compounds were searched from PubChem (28) and the MPD3 database (29). Their energy was minimized and they were stored in the “.mdb” format database. From the protein data bank (PDB), the SARS-CoV-2 3D main protease (3CL^pro^) structure was copied (30) and processed for energy minimization and missing hydrogen atoms. Finally, the rigid docking algorithm of MOE was applied and the prepared lipids' database was screened against the ligand-binding site of SARS-CoV-2 3CL^pro^. PyMOL was used for the visualization of docked complexes (31).

### Statistical analysis

The OmicShare tools (www.omicshare.com/tools) were used for heat map, hierarchical cluster, and principal component analysis (PCA) by using the hcluster algorithm as performed before (32). The peak area (from each variety with three replications) was used to perform the PCA and hierarchical cluster analysis (HCA) Data pre-processing was normalized and further details of normalization are represented (https://www.omicshare.com/forum/thread-407-1-1.html) a publically available online platform.

## Results

### Structure of major lipids compound

**Figure 1:**
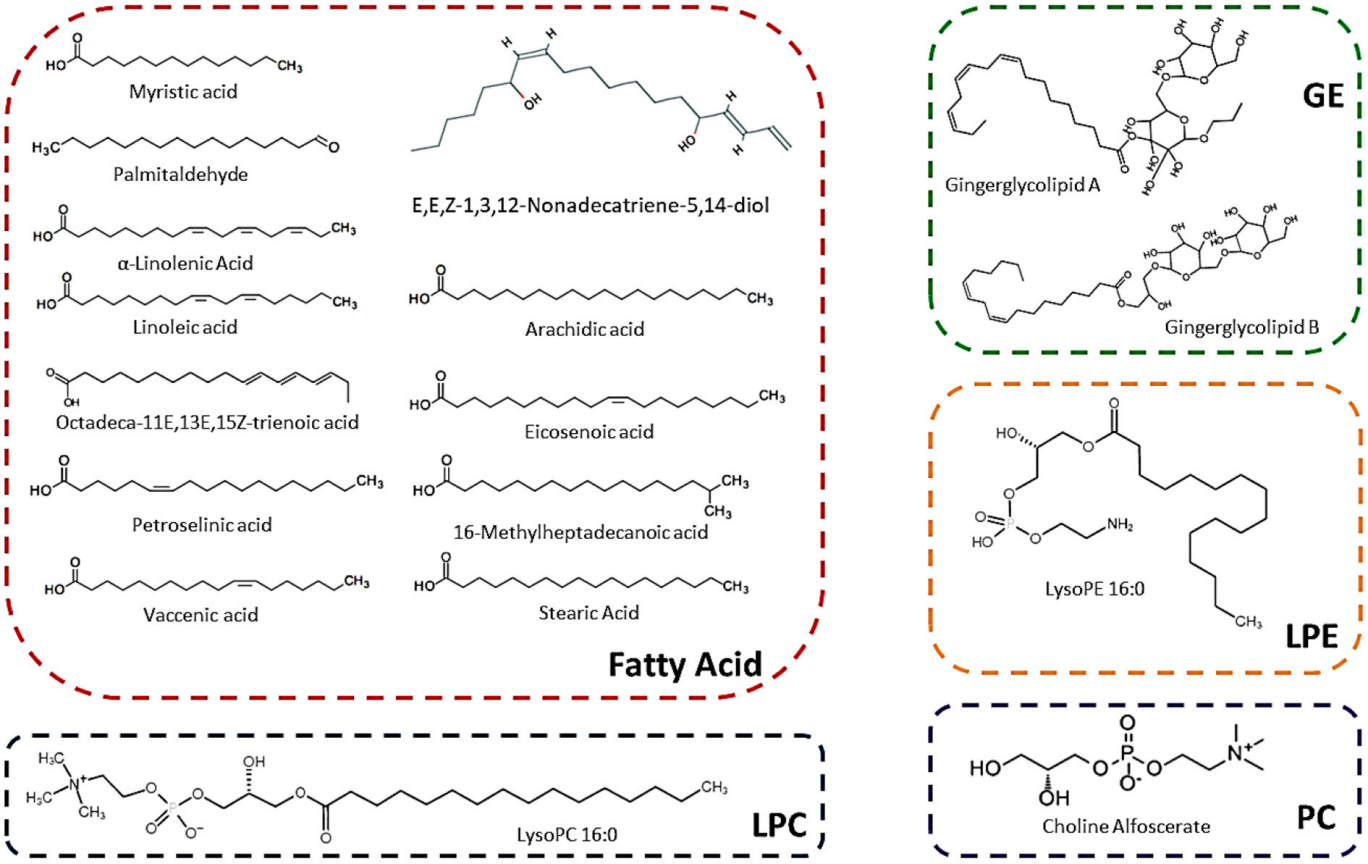
Structure of main lipids that are abundantly found in the rind of six sugarcane genotypes.

A total number of 134 lipids were identified from six sugarcane genotypes. These compounds belong to different sub-categories of lipids such as fatty acids, sphingolipids, phosphatidylcholine (PC), lysophosphatidylcholines (LPC), lysophosphatidylethanolamines (LPE), and glycerol esters (GE) (Table 1). The chemical formula, precursor ion Q1 (Da), daughter ion Q3 (Da), CAS-number, molecular weight, and ionization mode of lipids are present in Table 1. Among 134 lipid metabolism compounds, 119 compounds were first time identified in sugarcane genotypes (Table 1). Seventeen lipids compounds (including 12 fatty acids, 2 glycerol lipids, 1 LPC, 1 LPE, and 1 PC) were uniquely high in the rind of all sugarcane genotypes, among them, 11 compounds were reported first time in sugarcane rind (Figure 1; Table 1). The chemical structure of these 17 lipid compounds is represented (Figure 1). Among 17 major lipid compounds, myristic acid, linoleic acid, stearic acid, α-linolenic acid, arachidic acid, and petroselinic acid were reported before whereas the other compounds are reported first time in the rind of six sugarcane genotypes (Figure 1). Among 23 glycerol ester compounds (Table 1), gingerglycolipid A and gingerglycolipid B were uniquely high in the rind and these compounds are famous due to their promising pharmacological value (Figure 1).

**Table 1:** Details of lipid compounds identified in sugarcane genotypes.

**Sr. no**	**Compound Name**	**Class of compound**	**Molecular weight**	**Formula**	**Ionization mode**	**Q1 (Da)**	**Q3 (Da)**	**CAS**
1	#Undecylic Acid	Free fatty acids	186.162	C11H22O2	[M - H]-	185.15	185.16	112-37-8
2	Dodecanoic acid (Lauric acid)	Free fatty acids	200.178	C12H24O2	[M - H]-	199.17	199.17	143-07-7
3	#12-Oxo-10E-Dodecenoic Acid	Free fatty acids	212.141	C12H20O3	[M - H]-	211.13	183.14	65410-38-0
4	#12-Hydroxydodecanoic acid	Free fatty acids	216.173	C12H24O3	[M - H]-	215.17	169.16	505-95-3
5	#Myristoleic acid	Free fatty acids	226.193	C14H26O2	[M - H]-	225.19	207	544-64-9
6	#2-Dodecenedioic acid	Free fatty acids	228.136	C12H20O4	[M - H]-	227.13	183.14	6402-36-4
7	Myristic Acid	Free fatty acids	228.209	C14H28O2	[M - H]-	227.2	227.2	544-63-8
8	#Palmitaldehyde	Free fatty acids	240.245	C16H32O	[M - H]-	239	223	629-80-1
9	Pentadecanoic Acid	Free fatty acids	242.225	C15H30O2	[M - H]-	241.22	241.22	1002-84-2
10	Palmitoleic Acid*	Free fatty acids	254.225	C16H30O2	[M - H]-	253.22	235.19	373-49-9
11	#(7Z)-Hexadecenoic acid*	Free fatty acids	254.225	C16H30O2	[M - H]-	253.22	235.21	2416-19-5
12	Palmitic acid	Free fatty acids	256.24	C16H32O2	[M - H]-	255.23	237.22	57-10-3
13	#Choline Alfoscerate (glycerophosphocholine)	PC	257.103	C8H20NO6P	[M + H]+	258.11	104.11	28319-77-9
14	#10-Heptadecenoic Acid	Free fatty acids	268.24	C17H32O2	[M - H]-	267.23	267.23	29743-97-3
15	#Hexadecylsphingosine	Sphingolipids	273.267	C16H35NO2	[M + H]+	274.27	256.27	-
16	#Punicic acid (9Z,11E,13Z-octadecatrienoic acid)	Free fatty acids	278.225	C18H30O2	[M + H]+	279.23	95.1	544-72-9
17	#γ-Linolenic Acid*	Free fatty acids	278.225	C18H30O2	[M - H]-	277.22	277.22	506-26-3
18	α-Linolenic Acid*	Free fatty acids	278.225	C18H30O2	[M - H]-	277.22	277.22	463-40-1
19	#Octadeca-11E,13E,15Z-trienoic acid	Free fatty acids	278.225	C18H30O2	[M + H]+	279.2	149.4	25575-00-2
20	#Crepenynic acid	Free fatty acids	278.225	C18H30O2	[M - H]-	277.22	59.01	2277-31-8
21	#(9Z,11E)-Octadecadienoic acid	Free fatty acids	280.24	C18H32O2	[M - H]-	279.23	59.01	2540-56-9
22	Linoleic acid	Free fatty acids	280.24	C18H32O2	[M - H]-	279.23	279.23	60-33-3
23	#Oleamide (9-Octadecenamide)	Free fatty acids	281.272	C18H35NO	[M + H]+	282.28	247.24	301-02-0
24	Petroselinic acid*	Free fatty acids	282.256	C18H34O2	[M - H]-	281.25	281.25	593-39-5
25	#11-Octadecanoic acid(Vaccenic acid)*	Free fatty acids	282.256	C18H34O2	[M - H]-	281.25	281.25	506-17-2
26	#16-Methylheptadecanoic acid	Free fatty acids	284.272	C18H36O2	[M - H]-	283.26	283.26	2724-58-5
27	Stearic Acid	Free fatty acids	284.272	C18H36O2	[M - H]-	283.26	283.26	57-11-4
28	#1,18-Octadecanediol	Free fatty acids	286.287	C18H38O2	[M + H]+	287.29	97.2	3155-43-9
29	#4-Oxo-9Z,11Z,13E,15E-Octadecatetraenoic Acid	Free fatty acids	290.188	C18H26O3	[M + H]+	291	190.9	-
30	#12-Oxo-phytodienoic acid	Free fatty acids	292.204	C18H28O3	[M - H]-	291.2	235.17	85551-10-6
31	#13-Hydroxy-6,9,11-octadecatrienoic acid	Free fatty acids	294.219	C18H30O3	[M - H]-	293.21	193.1	74784-20-6
32	#13S-Hydroxy-9Z,11E,15Z-octadecatrienoic acid	Free fatty acids	294.219	C18H30O3	[M - H]-	293.21	195.14	87984-82-5
33	#E,E,Z-1,3,12-Nonadecatriene-5,14-diol	Free fatty acids	294.256	C19H34O2	[M - H]-	293.25	293.25	-
34	#9S-Hydroxy-10E,12Z-octadecadienoic acid*	Free fatty acids	296.235	C18H32O3	[M - H]-	295.23	195.14	15514-85-9
35	#13(S)-HODE;13(S)-Hydroxyoctadeca-9Z,11E-dienoic acid*	Free fatty acids	296.235	C18H32O3	[M - H]-	295.23	195.14	10219-69-9
36	#12,13-Epoxy-9-Octadecenoic Acid*	Free fatty acids	296.235	C18H32O3	[M - H]-	295.23	195.2	6799-85-5
37	#9(10)-EpOME;(9R,10S)-(12Z)-9,10-Epoxyoctadecenoic acid*	Free fatty acids	296.235	C18H32O3	[M - H]-	295.23	171.1	16833-56-0
38	Ricinoleic acid	Free fatty acids	298.251	C18H34O3	[M - H]-	297.24	183.14	141-22-0
39	1-Eicosanol	Free fatty acids	298.324	C20H42O	[M - H]-	297.32	183.1	629-96-9
40	#2R-Hydroxyoctadecanoic Acid	Free fatty acids	300.266	C18H36O3	[M - H]-	299.26	253.26	26633-48-7
41	3-Hydroxyoctadecanoic Acid	Free fatty acids	300.267	C18H36O3	[M - H]-	299.26	299.26	45261-96-9
42	Eicosadienoic acid	Free fatty acids	308.272	C20H36O2	[M - H]-	307.26	307.3	5598-38-9
43	#9-Hydroperoxy-10E,12,15Z-octadecatrienoic acid	Free fatty acids	310.1	C18H30O4	[M - H]-	309.21	209.1	111004-08-1
44	#13S-Hydroperoxy-6Z,9Z,11E-octadecatrienoic acid	Free fatty acids	310.214	C18H30O4	[M - H]-	309	209	121107-97-9
45	#Eicosenoic acid	Free fatty acids	310.287	C20H38O2	[M - H]-	309.28	309.28	26764-41-0
46	#5S,8R-DiHODE; (5S,8R,9Z,12Z)-5,8-Dihydroxyoctadeca-9,12-dienoate	Free fatty acids	312.23	C18H32O4	[M - H]-	311.22	223.17	-
47	#7S,8S-DiHODE; (9Z,12Z)-(7S,8S)-Dihydroxyoctadeca-9,12-dienoic acid	Free fatty acids	312.23	C18H32O4	[M - H]-	311.22	249.22	143288-65-7
48	#9-Hydroxy-13-oxo-10-octadecenoic Acid	Free fatty acids	312.23	C18H32O4	[M - H]-	311.22	293.21	-
49	#13S-Hydroperoxy-9Z,11E-octadecadienoic acid	Free fatty acids	312.23	C18H32O4	[M - H]-	311.1	171.3	33964-75-9
50	#9-Hydroxy-12-oxo-15(Z)-octadecenoic acid	Free fatty acids	312.23	C18H32O4	[M - H]-	311.22	223.17	-
51	Arachidic acid	Free fatty acids	312.303	C20H40O2	[M - H]-	311.3	183.1	506-30-9
52	#12,13-DHOME; (9Z)-12,13-Dihydroxyoctadec-9-enoic acid	Free fatty acids	314.246	C18H34O4	[M - H]-	313.24	251.24	263399-35-5
53	#9,12,13-Trihydroxy-10,15-octadecadienoic acid	Free fatty acids	328.225	C18H32O5	[M - H]-	327.22	291.2	-
54	#Cis-4,7,10,13,16,19-Docosahexaenoic Acid	Free fatty acids	328.24	C22H32O2	[M - H]-	327.23	283.3	6217-54-5
55	#9,10-Dihydroxy-12,13-epoxyoctadecanoic acid	Free fatty acids	330.241	C18H34O5	[M - H]-	329.23	199.13	-
56	#9,12,13-TriHOME; 9(S),12(S),13(S)-Trihydroxy-10(E)-octadecenoic acid	Free fatty acids	330.241	C18H34O5	[M - H]-	329.23	211.14	97134-11-7
57	#9,10,13-Trihydroxy-11-Octadecenoic Acid	Free fatty acids	330.241	C18H34O5	[M - H]-	329.23	229.15	29907-57-1
58	#Monopalmitin	Glycerol ester	330.277	C19H38O4	[M + H]+	331.28	313.27	542-44-9
59	#1-α-Linolenoyl-glycerol	Glycerol ester	352.261	C21H36O4	[M + H]+	353.27	261.22	-
60	#2-α-Linolenoyl-glycerol	Glycerol ester	352.261	C21H36O4	[M + H]+	353.27	261.22	55268-58-1
61	#2-Linoleoylglycerol	Glycerol ester	354.277	C21H38O4	[M + H]+	355.28	263.24	3443-82-1
62	#1-Linoleoylglycerol	Glycerol ester	354.277	C21H38O4	[M + H] +	355.28	263.24	2277-28-3
63	#1-Oleoyl-Sn-Glycerol	Glycerol ester	356.293	C21H40O4	[M + H]+	357.3	265.4	129784-87-8
64	#1-O-Caffeoyl-3-O-p-coumaroylglycerol	Glycerol ester	400.116	C21H20O8	[M - H]-	399.11	163.04	-
65	#LysoPE 14:0	LPE	425.254	C19H40NO7P	[M + H]+	426.26	285.24	-
66	#1-O-Feruloyl-3-O-caffeoylglycerol	Glycerol ester	430.126	C22H22O9	[M - H]-	429.12	429.12	-
67	#LysoPE 15:0(2n isomer)	LPE	439.27	C20H42NO7P	[M + H]+	440.28	299.26	-
68	#LysoPE 15:0	LPE	439.27	C20H42NO7P	[M + H]+	440.28	299.26	-
69	#LysoPE 16:1	LPE	451.27	C21H42NO7P	[M + H]+	452.28	311.26	-
70	#LysoPE 16:0(2n isomer)	LPE	453.286	C21H44NO7P	[M + H]+	454.29	313.27	-
71	#LysoPE 16:0	LPE	453.286	C21H44NO7P	[M + H]+	454.29	313.27	53862-35-4
72	#LysoPE 17:1	LPE	465.286	C22H44NO7P	[M + H]+	466.29	325.27	-
73	#LysoPE 17:1(2n isomer)	LPE	465.286	C22H44NO7P	[M + H]+	466.29	325.27	-
74	#LysoPE 17:0	LPE	467.301	C22H46NO7P	[M + H]+	468.31	327.29	-
75	#LysoPC 14:0	LPC	467.301	C22H46NO7P	[M + H]+	468.31	184.07	20559-16-4
76	#LysoPE 18:3(2n isomer)	LPE	475.27	C23H42NO7P	[M + H]+	476.28	335.26	-
77	#LysoPE 18:3	LPE	475.27	C23H42NO7P	[M + H]+	476.28	335.26	-
78	#LysoPE 18:2(2n isomer)	LPE	477.286	C23H44NO7P	[M + H]+	478.29	337.27	-
79	#LysoPE 18:2	LPE	477.286	C23H44NO7P	[M + H]+	478.29	337.27	-
80	#LysoPE 18:1(2n isomer)	LPE	479.301	C23H46NO7P	[M + H]+	480.31	339.29	-
81	#LysoPE 18:1	LPE	479.301	C23H46NO7P	[M + H]+	480.31	339.29	89576-29-4
82	#LysoPC 15:1	LPC	479.301	C23H46NO7P	[M + H]+	480.31	184.07	-
83	#LysoPC 15:0(2n isomer)	LPC	481.317	C23H48NO7P	[M + H]+	482.32	184.07	-
84	#LysoPE 18:0(2n isomer)	LPE	481.317	C23H48NO7P	[M + H]+	482.32	341.31	-
85	#LysoPE 18:0	LPE	481.317	C23H48NO7P	[M + H]+	482.32	341.31	69747-55-3
86	#LysoPC 15:0	LPC	481.317	C23H48NO7P	[M + H]+	482.32	184.07	108273-89-8
87	#LysoPG 16:1	Glycerol ester	482.262	C22H43O9P	[M - H]-	481.25	245.04	-
88	#LysoPG 16:0	Glycerol ester	484.278	C22H45O9P	[M - H]-	483.27	255.23	-
89	#LysoPC 16:2	LPC	491.301	C24H46NO7P	[M + H]+	492.31	184.07	-
90	#LysoPC 16:2(2n isomer)	LPC	491.301	C24H46NO7P	[M + H]+	492.31	184.07	-
91	#LysoPC 16:1(2n isomer)	LPC	493.317	C24H48NO7P	[M + H]+	494.32	184.07	-
92	#LysoPC 16:1	LPC	493.317	C24H48NO7P	[M + H]+	494.32	184.07	76790-27-7
93	#LysoPC 16:0	LPC	495.332	C24H50NO7P	[M + H]+	496.34	184.07	17364-16-8
94	#LysoPC 16:0(2n isomer)	LPC	495.332	C24H50NO7P	[M + H]+	496.34	184.07	-
95	#LysoPE 20:3	LPE	503.301	C25H46NO7P	[M + H]+	504.31	363.29	-
96	#LysoPE 20:3(2n isomer)	LPE	503.301	C25H46NO7P	[M + H]+	504.31	363.29	-
97	#LysoPE 20:2	LPE	505.317	C25H48NO7P	[M + H]+	506.32	365.31	-
98	#LysoPE 20:2(2n isomer)	LPE	505.317	C25H48NO7P	[M + H]+	506.32	365.31	-
99	#LysoPC 17:2	LPC	505.317	C25H48NO7P	[M + H]+	506.32	184.07	-
100	#LysoPC 17:1	LPC	507.332	C25H50NO7P	[M + H]+	508.34	184.07	-
101	#LysoPC 17:0	LPC	509.348	C25H52NO7P	[M + H]+	510.36	184.07	50930-23-9
102	#LysoPC 17:0(2n isomer)	LPC	509.348	C25H52NO7P	[M + H]+	510.36	184.07	-
103	#2-α-Linolenoyl-glycerol-1-O-glucoside	Glycerol ester	514.314	C27H46O9	[M + H]+	515.32	261.22	-
104	#1-α-Linolenoyl-glycerol-3-O-glucoside	Glycerol ester	514.314	C27H46O9	[M + H]+	515.32	261.22	-
105	#2-Linoleoylglycerol-1-O-glucoside	Glycerol ester	516.33	C27H48O9	[M + H]+	517.34	263.24	-
106	#LysoPC 18:3	LPC	517.317	C26H48NO7P	[M + H]+	518.32	184.07	-
107	#LysoPC 18:3(2n isomer)	LPC	517.317	C26H48NO7P	[M + H]+	518.32	184.07	-
108	#LysoPC 18:2(2n isomer)	LPC	519.332	C26H50NO7P	[M + H]+	520.34	184.07	-
109	#LysoPC 18:2	LPC	519.332	C26H50NO7P	[M + H]+	520.34	184.07	-
110	#PS(18:2)	Glycerol ester	521.275	C24H44NO9P	[M - H]-	520.27	153	-
111	#LysoPC 18:1	LPC	521.348	C26H52NO7P	[M + H]+	522.36	184.07	-
112	#LysoPC 18:1(2n isomer)	LPC	521.348	C26H52NO7P	[M + H]+	522.36	184.07	-
113	#LysoPC 18:0(2n isomer)	LPC	523.364	C26H54NO7P	[M + H]+	524.37	184.07	-
114	#LysoPC 18:0	LPC	523.364	C26H54NO7P	[M + H]+	524.37	184.07	19420-57-6
115	#LysoPC 19:2(2n isomer)	LPC	533.348	C27H52NO7P	[M + H]+	534.36	184.07	-
116	#LysoPC 19:2	LPC	533.348	C27H52NO7P	[M + H]+	534.36	184.07	-
117	LysoPC 19:1	LPC	535.364	C27H54NO7P	[M + H]+	536.37	184.07	-
118	#LysoPC 20:4	LPC	543.332	C28H50NO7P	[M + H]+	544.34	184.07	-
119	#LysoPC 20:3	LPC	545.348	C28H52NO7P	[M + H]+	546.36	184.07	1199257-41-4
120	#LysoPC 20:2	LPC	547.364	C28H54NO7P	[M + H]+	548.37	184.07	-
121	#LysoPC 20:2(2n isomer)	LPC	547.364	C28H54NO7P	[M + H]+	548.37	184.07	-
122	LysoPC 20:1	LPC	549.379	C28H56NO7P	[M + H]+	550.39	184.07	-
123	#PE(oxo-11:0/16:0)	Glycerol ester	635.414	C32H62NO9P	[M + H]+	636.41	495.4	-
124	#1-(9Z-Octadecenoyl)-2-(9-oxo-nonanoyl)-sn-glycero-3-phosphocholine	Glycerol ester	675.448	C35H66NO9P	[M + H]+	676.45	184.08	-
125	#1-Linolenoyl-rac-glycerol-diglucoside	Free fatty acids	676.367	C33H56O14	[M + H]+	677.37	677.37	-
126	#Gingerglycolipid A	Glycerol ester	676.367	C33H56O14	[M - H]-	675.36	397.14	145937-22-0
127	#2-α-Linolenoyl-glycerol-1,3-di-O-glucoside	Glycerol ester	676.367	C33H56O14	[M + H]+	677.37	261.22	-
128	#1-α-Linolenoyl-glycerol-2,3-di-O-glucoside	Glycerol ester	676.367	C33H56O14	[M + H]+	677.37	261.22	-
129	#2-Linoleoylglycerol-1,3-di-O-glucoside	Glycerol ester	678.383	C33H58O14	[M + H]+	679.39	263.24	-
130	#1-Linoleoyl-sn-glycerol-diglucoside	Free fatty acids	678.383	C33H58O14	[M + H]+	679.39	263.24	-
131	#1-Linoleoylglycerol-2,3-di-O-glucoside	Glycerol ester	678.383	C33H58O14	[M + H]+	679.39	263.24	-
132	#Gingerglycolipid B	Glycerol ester	678.383	C33H58O14	[M - H]-	677.38	397.13	88168-90-5
133	#Gingerglycolipid C	Glycerol ester	680.398	C33H60O14	[M - H]-	679.39	397.13	-
134	#PC(oxo-11:0/18:2)	PC	701.46	C37H68NO9P	[M + H]+	702.46	184.07	-

^*^Represents isomers; #means first time reported in sugarcane rind; CAS, Chemical Abstracts Service.

### Principal component analysis of lipids compounds

**Figure 2:**
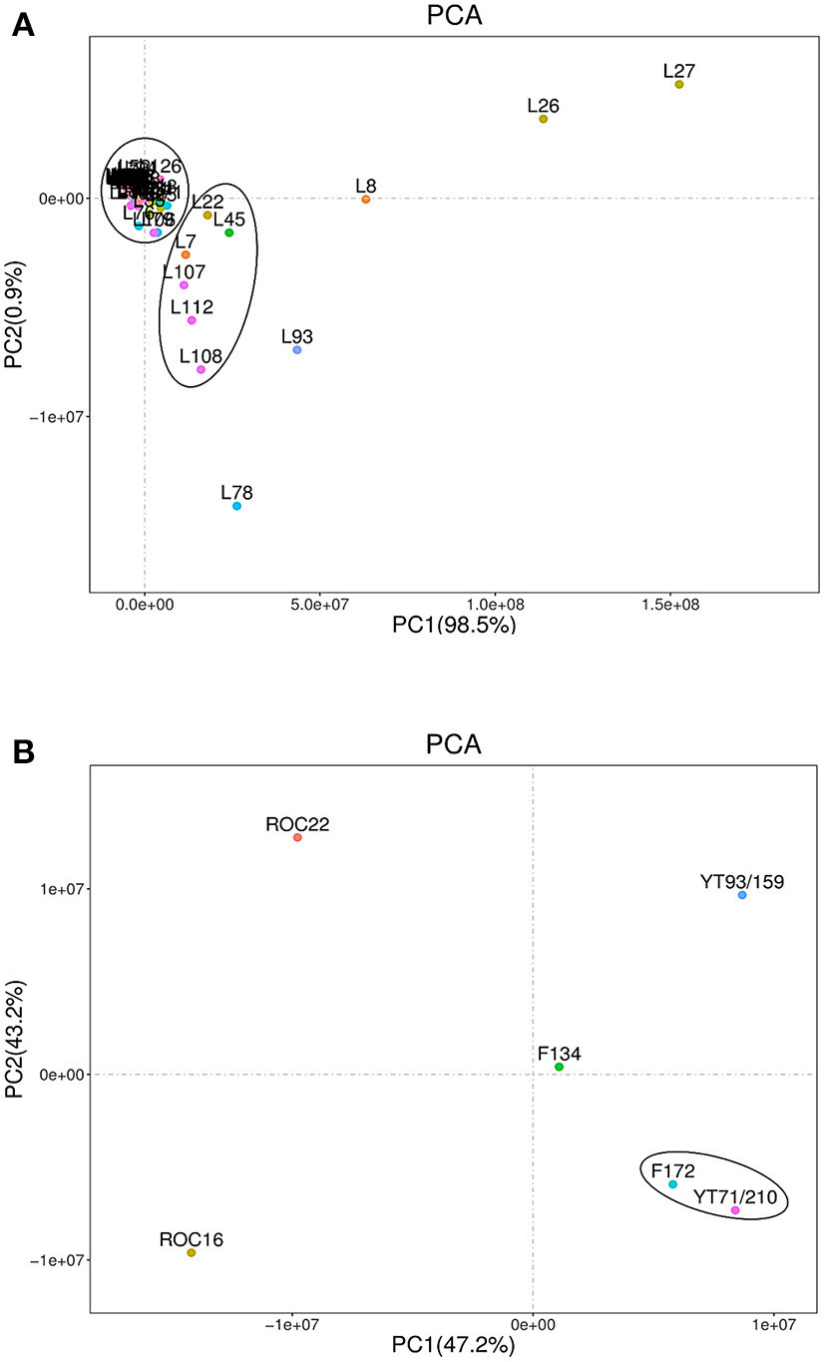
Principal component analyses of lipid compounds among six sugarcane genotypes. Each variety represents the data of three biological repeats. **(A)** Represents the metabolic-wise PCA (the L1-L134 numbers are corresponding to the sequence of lipid compounds in Table 1). **(B)** Represents the variety-wise PCA.

The sugarcane variety-wise and individual lipid compounds-wise PCA (scatter plot) were performed (Figure 2). The lipids scatter plot was achieved through PCA (Figure 2A). In the scatter plot the lipids compounds showed two distinct clusters (Figure 2A). Most of the lipids compounds gathered in one cluster on the intersection point of the x-axis and y-axis while the second group (includes 6 lipid compounds) was clustered slightly below the first cluster (Figure 2A). The five lipid compounds including palmitaldehyde (L8), 16-Methylheptadecanoic acid (L26), stearic acid (L27), lysoPE 18:2 (2n isomer) (L78), and lysoPC 16:0 (L93) didn't fall in any cluster and showed their unique identity on the PCA scatter plot (Figure 2A). These five lipid compounds revealed maximum divergent appearances on PCA and displayed the highest absolute scores values in both PC1 98.5% (on the x-axis) and PC2 0.9% (on the y-axis) (Figure 2A). Moreover, these compounds were reported first time in this study except for stearic acid.

Sugarcane variety-wise scattered plot was attained using PCA (Figure 2B). The variety-wise PCA revealed that the F172 and YT71/210 varieties were very close to each other and made their distinct group from the other four varieties (Figure 2B). The other four sugarcane varieties such as F134, YT93/159, ROC16, and ROC22 were far away from each other and showed their distinct characteristics on scattered PCA plots and displayed higher absolute scores on both x-axis (PC1) and y-axis (PC2) (Figure 2B). Also, these four varieties were far away from F172 and YT71/210 clusters (Figure 2B). The PC1 accounted for 47.2% while the PC2 accounted for 43.2% of absolute scores values on variety-wise PCA scattered plots (Figure 2B). These outcomes revealed that most of the sugarcane genotypes bear unique characteristics regarding lipid metabolism and these results are interesting for the researchers and breeders who are engaged with lipid metabolism (sugarcane breeding programs) for enhancing the biodiesel and bioethanol production from sugarcane residues (such as rind) (33). Additionally, the lipid compounds have promising effects on the skin and have unique pharmacological effects (34, 35). Exploring the lipid metabolism of sugarcane will help to know more compounds that have promising cosmetic and pharmacological value.

### Hierarchical cluster analysis of lipids from rind samples

**Figure 3:**
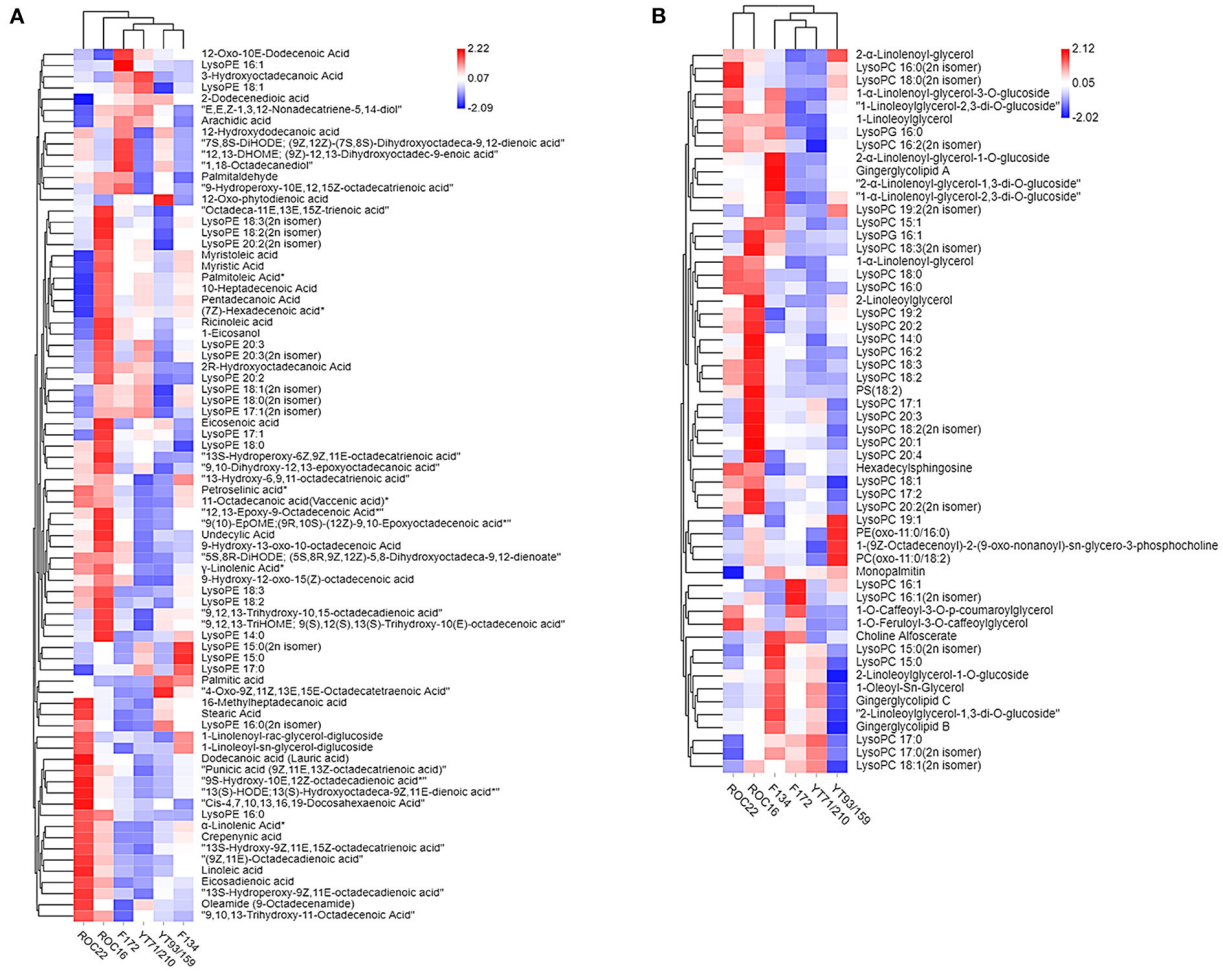
Hierarchical cluster analysis represents the distribution of lipid compounds in different sugarcane genotypes. The row represents lipid compounds and the column represents sugarcane genotypes. **(A)** Fatty acid and LPE. **(B)** LPC, PC, Glycerol ester, and hexadecylsphingosine. Red indicates high concentration and blue color means low concentration. Each variety represents the data of three biological repeats.

The heat map and hierarchical cluster analysis (HCA) of lipid compounds in the rind of six sugarcane genotypes are characterized (Figure 3). Figure 3A heat map represents the fatty acids and LPE compounds whereas the Figure 3B heat map signifies the LPC, PC, hexadecylsphingosine (sphingolipids), and glycerol ester compounds. The HCA analysis revealed that the YT71/210 and F172 stand near to each other and fall in the same group (Figure 3A). The F134 and YT93/159 are very close to each other and made the same cluster (Figure 3A). Additionally, the other two varieties ROC22 and ROC16 didn't make any cluster and represent their unique characteristics in hierarchical cluster analysis (Figure 3A); however, they were closer to each other (Figure 3A). According to the abundance of individual fatty acids and LPE compounds, all LPC and fatty acid compounds made two major groups and several sub-clusters in the rind of six sugarcane genotypes (Figure 3A). HCA analysis revealed that the ROC22 and ROC16 are closer to each other and fall in the same cluster whereas the F172 and YT71/210 again made the same cluster (Figure 3B) as in the first heat map (Figure 3A). Interestingly, the YT93/159 and F134 didn't make any clusters; it signifies that these two genotypes have unique characteristics (regarding LPC, PC, hexadecylsphingosine, and glycerol ester compounds) (Figure 3B).

Some high-value fatty acids such as lauric acid, linoleic acid, eicosadienoic acid (an omega-6 fatty acid), punicic acid (an omega-5 fatty acid), crepenynic acid, α-linolenic acid, stearic acid, 16-methylheptadecanoic acid, petroselinic acid were uniquely high in the rind of ROC22 (Figure 3A). α-Linolenic acid (which can't be synthesized by humans, is an essential fatty acid) have strong anti-arrhythmic properties and it is associated with cancer and cardiovascular disease (34). Similarly, linoleic acid has also promising pharmacological value especially related to cardiovascular, cardio-metabolic effects, and brain function (35). Most of the LPE compounds were high in the rind of ROC16 and ROC22 whereas some LPE such as LysoPE 16:1 and LysoPE 15:0 were higher in F172 and F134, respectively (Figure 3A).

Among the major glycerol esters such as gingerglycolipid A, gingerglycolipid B, and gingerglycolipid C were uniquely high in the rind of the F134 genotype whereas the YT93/159 has the lowest concentration of these glycerol esters (Figure 3B). These glycerol esters have strong anti-cancer effects and several other health-promoting properties (36). The PC (oxo-11:0/18:2) and PE (oxo-11:0/16:0) compounds were uniquely high in the rind of YT93/159 whereas the maximum level of choline alfoscerate [effective choline compound for Alzheimer's disease treatment, also it is the precursor of acetylcholine (20)] was observed in the of F134 followed by F172 (Figure 3B). Among all sugarcane genotypes, the ROC16 rind showed a higher level of LPC compounds followed by ROC22 (Figure 3B). In short, the rind of different cultivated sugarcane genotypes is a promising source of lipid compounds that have auspicious nutritive and pharmacological value; however, the amount of these compounds varies broadly between sugarcane genotypes. Commonly, sugarcane rind is utilized for electricity generation, wasted, or used as organic fertilizer; however, we have reported several lipid compounds in sugarcane rind that have significant nutritive, cosmetic, and pharmacological value. These compounds can be extracted from the sugarcane rind to fulfill the increasing nutritive demands and can be used in the pharmacological and cosmetic industries.

The PCA and HCA results together revealed that the F172 and YT71/210 genotypes have quite similar levels of lipid compounds because in the PCA and HCA analysis they stand very close to each other and made almost the same cluster (Figures 2, 3). Besides this, the HCA and PCA results represented that the F134, YT93/159 group and ROC22, ROC16 group bears their unique characteristics regarding lipids compounds (Figures 2, 3); however, the F134, YT93/159 group and ROC22, ROC16 group are quite similar regarding fatty acid, LPE and LPC, PC, glycerol ester, and hexadecylsphingosine compounds in the first and second heat-map, respectively, (Figures 3A,B).

### Sugarcane lipids biosynthesis pathway and transcriptomic results

**Figure 4:**
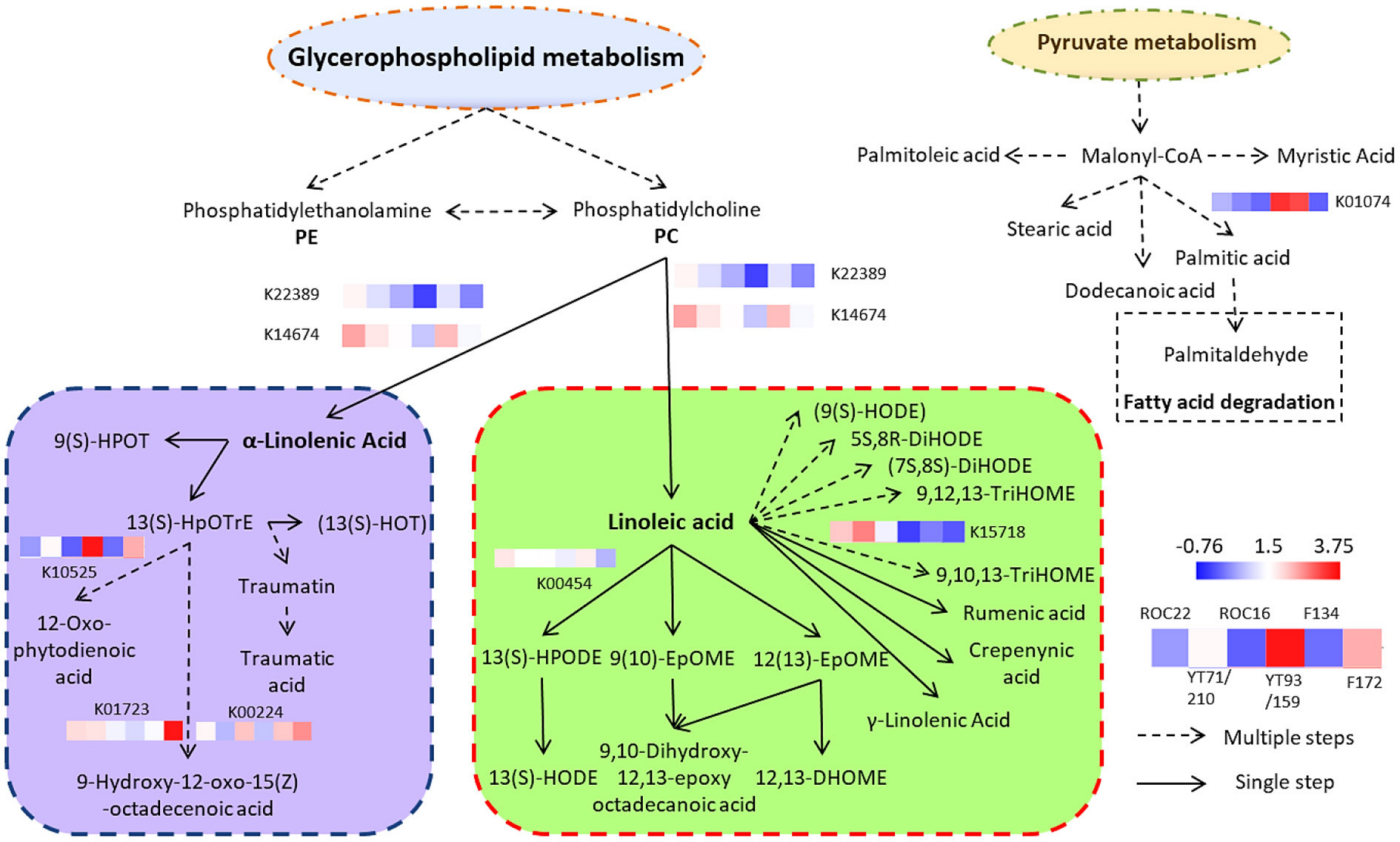
Complex sugarcane lipids biosynthesis pathway (thru Glycerophospholipid and Pyruvate metabolism) and expression of DEGs in the rind of six sugarcane genotypes. Each expression level is the mean of three biological repeats (the raw transcriptomic data is represented in Supplementary File 1).

The sugarcane lipid biosynthesis pathway is characterized (Figure 4). In sugarcane, the lipid compounds are biosynthesized through glycerophospholipid and pyruvate metabolism. From glycerophospholipid metabolism, glycerophospholipids are formed which biosynthesized phosphatidylcholine (PC) (Figure 4). By the activity of the TAG lipase (TGL4) enzyme, the PC is concerted into α-linolenic acid and linoleic acid which are famous omega-3 and −6 fatty acids, respectively (Figure 4). The α-linolenic acid (essential fatty acids) further biosynthesized complex fatty acids conjugates and fatty acyls such as 13(s)-HpOTrE and 12-Oxo-phytodienoic acid, respectively (Figure 4). These two fatty acids are major fatty acids of sugarcane (Figure 1) and they are precursors of several fatty acyls, fatty acids conjugates, and octadecanoid (Figure 4). The pyruvate metabolism produces malonyl-CoA which further biosynthesized (after several steps) diverse groups of saturated fatty acids such as stearic acid, myristic acid, dodecanoic acid (lauric acid), palmitic acid, and some saturated fatty acids such as palmitoleic acid (Figure 4). These all saturated and unsaturated fatty acids are considered phytocompounds and their presence restrict microbial activity (18). From palmitic acid, palmitaldehyde is formed by the fatty acid degradation pathway (Figure 4).

The transcriptomic results revealed that eight genes were differentially expressed in the rind of six sugarcane genotypes associated with the α-linolenic acid and linoleic acid pathway (Figure 4). The α-linolenic acid and linoleic acid biosynthesis genes such as K22389 and K14674 were highly expressed in the rind of ROC22 followed by F134 whereas the YT93/159 revealed the lowest expression of these genes (Figure 4; Supplementary File 1). The metabolic results also showed that the rind of ROC22 has the highest level of α-linolenic acid and linoleic acid (Figure 3). The palmitic acid biosynthesis gene K01074 was expressed higher in the rind of YT93/159 and F134 followed by ROC22 whereas the other genotypes showed the lowest expression of this gene (Figure 4). The HCA analysis determined that the palmitic acid level was highest in YT93/159 followed by F134 (Figure 3). The expressions of K22389, K14674, and K01074 genes are highly correlated with the metabolic results (Figures 3, 4) which will beneficial for further study on the biosynthesis pathways of lipids in sugarcane.

### Therapeutic potential of lipids against SARS-CoV-2

**Figure 5:**
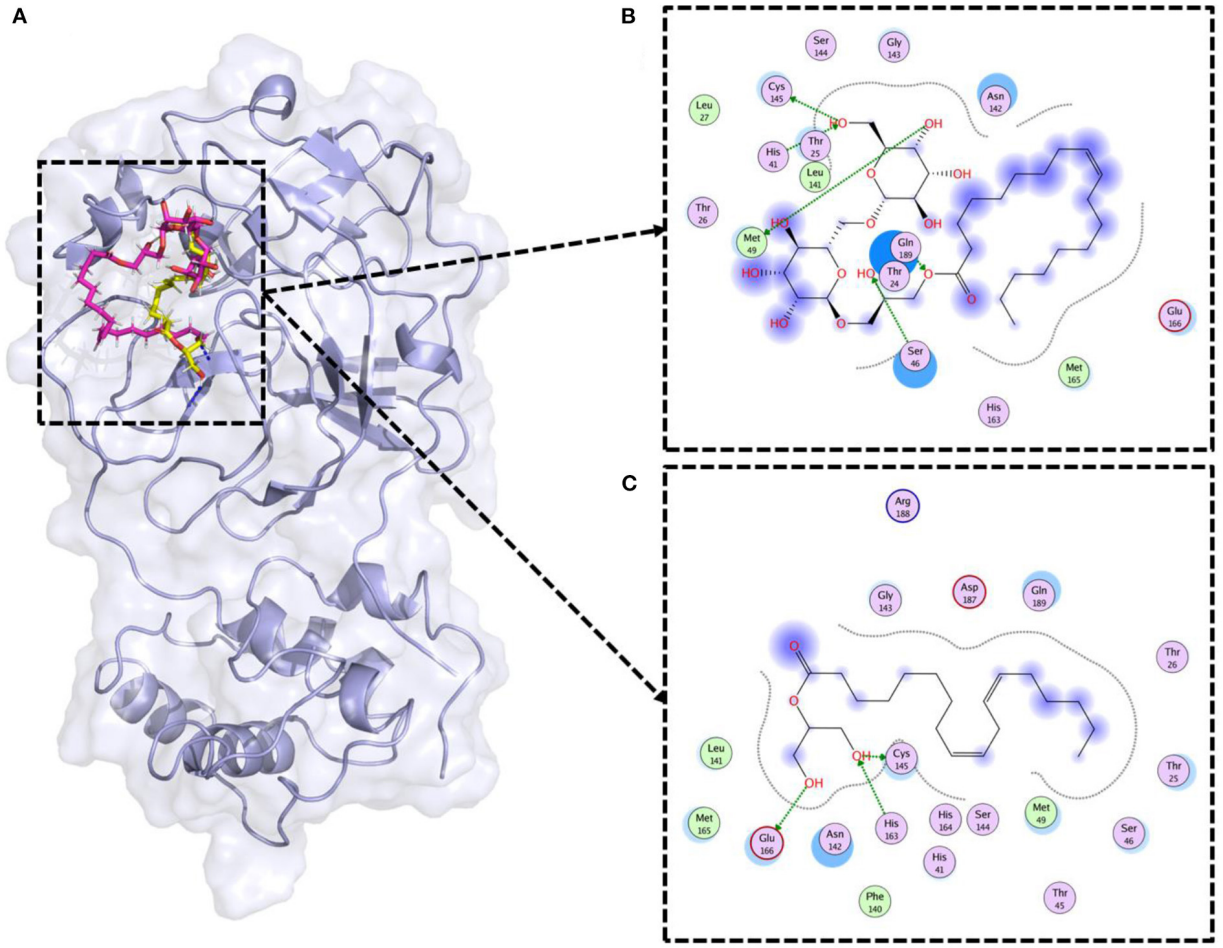
Molecular docking of lipid compounds against SARS-CoV-2 main protease. **(A)** 3D structural representation of top lipids bound 3CL^pro^ (PDB ID: 6LU7). The pink color ligand is Gingerglycolipid C, while the yellow color ligand is 2-Linoleoylglycerol. **(B)** Binding mode of Gingerglycolipid C inside the binding pocket of 3CL^pro^. **(C)** Binding mode of 2-Linoleoylglycerol inside the binding pocket of 3CL^pro^.

Molecular docking is a widely used computational approach to study the ligand binding dynamics with the protein (37). Lipid compounds therapeutic potential was assessed through molecular docking. SARS-CoV-2 3CL^pro^ was the target in this study (PDB ID: 6LU7). The 3CL^pro^ is vital for SARS-CoV-2 replication and inhibition of this enzyme can help to fight against COVID-19 (26). The lipids' were docked against the 3D structure of 3CL^pro^ (Figure 5A). Previous studies reported that Cys-His catalytic dyad (Cys145 and His41) is the vital residue to target the 3CL^pro^ (26, 38). Therefore, only those compounds were selected that were making strong binding interactions with the Cys145 and His41 residues. Results revealed that two compounds (Gingerglycolipid C and 2-Linoleoylglycerol) as potential therapeutic candidates for SARS-CoV-2 (Figure 5). Gingerglycolipid C bounds with 3CL^pro^ with −12.80 kcal/mol binding energy score and made strong hydrogen bindings with Cys145, His41, Ser46, and Met49 (Figure 5B). While, 2-Linoleoylglycerol bound with 3CL^pro^ having a −10.78 kcal/mol binding energy score and made strong hydrogen bindings with Cys145, His163, and Glu166, and strong polar interaction with His41 together with other binding site residues (Figure 5C).

## Discussion

The rind of sugarcane is a potential source for primary and secondary metabolites that have wide-ranging functions and are significant for cosmetic and pharmacological value (6, 39, 40). Lipids are an essential constituent of all plant cells (14, 15), providing a hydrophobic barrier that allows the separation among the subcellular structures of the cell. Lipids also activate several enzymes such as stearyl CoA desaturase, β-hydroxybutyric dehydrogenase, and glucose-6-phosphatase. Recently, it is revealed that lipids play a central role in cell membranes remodeling during drought and cold stress in *Arabidopsis thaliana* (41). Compared with bagasse, the sugarcane rind is mostly used to make hardboards, burned to produce energy, or as animal feed (7). In this study, we have reported new lipids compounds first time in sugarcane rinds and highlighted novel pharmaceutical values of sugarcane lipids that are worth paying attention for future lipidomics studies (Table 1).

The fatty acids and their derivatives are known for antifungal, antibacterial, and antimalarial activity (18, 42). Myristic acid has antifungal activity against several pathogenic funguses such as *Alternaria solani* (causing early blight disease in tomato and potato plants), *Penicillium italicum* (cause citrus blue mold), *Aspergillus niger, Fusarium oxysporum* (Causing Pokkah Boeng disease in sugarcane), *Emericella nidulans* (cause disease in humans), *Penicillium glabrum* and *Candida albicans* (43). In plants, linoleic acid (polyunsaturated fatty acid) has strong antifungal activity against various pathogenic fungi (43). The rind of ROC22 contains the highest level of linoleic acid and α-linolenic acid (essential fatty acids) whereas the F172 and YT71/210 possess the lowest level of these compounds (Figure 3).

Undecylic Acid can inhibit the activity of *Trichophyton rubrum* (a dermatophytic fungus that causes athlete's foot and ringworm) and *Saccharomyces cerevisiae* (44). Palmitic acid also showed resilient activity against *Alernaria* spp., *Cucumerinum lagenarium, Fusarium oxysporum*, and *Emericella nidulans* (43). Additionally, palmitic acid, linoleic acid, stearic acid, and linoleic acid help to acclimatize the salt stress in cucumber (45). These all fatty acids are abundantly present in the rind and are considered as major fatty acids of sugarcane (Figure 1). Some lipid compounds have promising effects on different human diseases. The lipid glycerol ester compounds such as gingerglycolipid A, gingerglycolipid B, and gingerglycolipid C have strong anti-ulcer and anti-tumor activities (13). The 2-Linoleoylglycerol can inhibit the activity of anandamide and 2-arachidonolyglycerol in humans (46). The rind of F134 showed a high concentration of gingerglycolipid A, B, and C followed by YT71/210 (Figure 3) whereas the ROC16 rind possesses a high level of 2-Linoleoylglycerol compound (Figure 3).

Gingerglycolipid C belongs to the class of organic compounds known as glycosylmonoacylglycerols. These glycosylglycerols form an ester linkage between the fatty acyl chain and the glycerol moiety. Previously, it has been reported that gingerglycolipid C compound has anti-tumor and anti-ulcer activities (13). Previously, it is reported that 2-linoleoylglycerol is a partial agonist at the human cannabinoid type 1 receptor and can modulate the activity of the established endocannabinoid (46). Phytochemicals such as 2-linoleoylglycerol from *Scolymus maculatus* L. have broad-spectrum antioxidant and antimicrobial activities to cure different diseases (47). 2-Linoleoylglycerol exhibits strong activity against *Salmonella typhimurium* (Gram-negative bacterium), *Staphylococcus aureus* (staphylococcal bacteria), and *Candida albicans* (cause fungal infection in humans) (47). Cultivated sugarcane showed dissimilar levels of therapeutic and medicinal lipid compounds such as ROC22 have a high level of essential fatty acid ‘α-linolenic acid' whereas the F134 and ROC16 showed a high level of lipid-glycerol ester compounds (Figure 3). The F134 and ROC16 rinds are better for therapeutic compounds, however, ROC22 rinds have a high level of essential fatty acid required for maintaining normal human health. In short, the lipid compounds have promising health-promoting activities that can be utilized as therapeutic agents to protect humans from several chronic.

Earlier studies have explored the potential therapeutic roles of lipids in human health (48). To access the pharmacological value of sugarcane lipid compounds we have examined their activity against SARS-CoV-2. COVID-19 (coronavirus disease 2019) pandemic caused by positive-stranded RNA virus SARS-CoV-2 (49), infected >583 million with >6.4 million people died worldwide according to the World Health Organization COVID-19 dashboard (https://covid19.who.int). Few vaccines are approved for COVID-19 but due to rapid mutations and novel variants, they are not fully effective against all types of SARS-CoV-2 infections. Herein, our results revealed two lipid compounds from sugarcane rind, gingerglycolipid C and 2-linoleoylglycerol, as potential SARS-CoV-2 therapeutic agents. Both compounds showed strong binding interactions with 3CL^pro^ of SARS-CoV-2 catalytic dyad residues Cys145 and His41. Gingerglycolipid C and 2-linoleoylglycerol compounds attained stable conformations inside the binding pocket of 3CL^pro^ with docking scores of−12.80 kcal/mol and−10.78 kcal/mol. Both compounds also have good drug-like properties. Thus, our findings warrant further *in vivo* and *in vitro* studies to explore their therapeutic potential in detail to fight against COVID-19.

## Conclusions

To conclude, we have reported 119 lipid compounds for the first time in the rind of six cultivated sugarcane genotypes. By using transcriptomic and metabolomic data, we have found that several genes associated with α-linolenic acid and linoleic acid biosynthesis were highly expressed in the rind of ROC22 whereas YT93/159 revealed the lowest expression. Among six sugarcane cultivars, the ROC22 and ROC16 showed a high level of lipid compounds. Moreover, we also found that the two lipid-glycerol ester compounds (2-linoleoylglycerol and gingerglycolipid C) have strong binding interactions with 3CL^pro^ of SARS-CoV-2 and these compounds can be potentially utilized as therapeutic agents against SARS-CoV-2. The rind of ROC16 revealed a high level of 2-linoleoylglycerol whereas the F134 rind showed a high concentration of gingerglycolipid C. In short, sugarcane is a storehouse of promising lipid compounds that have auspicious cosmetic and pharmacological value. Further studies should be conducted to screen more lipid-glycerol ester compounds and investigate their action as a therapeutic against COVID-19 in humans. Moreover, simplified extraction protocols should be developed to harvest more valuable compounds from sugarcane rinds.

## Data availability statement

The datasets presented in this study can be found in online repositories. The names of the repository/repositories and accession number(s) can be found below: NCBI with accession PRJNA824938.

## Author contributions

MR: conceptualization, project administration, visualization, and roles/writing–original draft. MR, QA, and MT: data curation. MR, DW, MT, and MD: formal analysis. LW: funding acquisition. MR, MD, LM, and MT: investigation. MR, MD, QA, and MT: methodology. MR, SH, LM, MD, and LW: resources. MR and MT: software. LH and LW: supervision. MR, MT, and MD: validation. MT, LH, and LW: writing–review and editing. All authors contributed to the article and approved the submitted version.

## Funding

This work was supported by the Natural Science Foundation of Guangxi (2020GXNSFDA238027), a Postdoctoral Project from GXU, Guangxi Natural Science Foundation Youth Fund (2022GXNSFBA035665), and the National Natural Science Foundation of China (31771775 and 31171524).

## Conflict of interest

The authors declare that the research was conducted in the absence of any commercial or financial relationships that could be construed as a potential conflict of interest.

## Publisher's note

All claims expressed in this article are solely those of the authors and do not necessarily represent those of their affiliated organizations, or those of the publisher, the editors and the reviewers. Any product that may be evaluated in this article, or claim that may be made by its manufacturer, is not guaranteed or endorsed by the publisher.

## Supplementary material

Supplementary File 1 Raw metabolomic and transcriptomic data.

Supplementary Figure 1 Representing the methodology used for the detection of different lipid compounds in the rind of six sugarcane varieties.
