# Supplementary material for: A high-throughput lipidomics and transcriptomic approach reveals novel compounds from sugarcane linked with promising therapeutic potential against COVID-19
Source: Front Nutr. 2022 Sep 2;9:988249. doi: 10.3389/fnut.2022.988249 (PMC9480494; doi:10.3389/fnut.2022.988249)
Supplement: Supplementary Figure 1 — Representing the methodology used for the detection of different lipid compounds in the rind of six sugarcane varieties. [file Table_1.DOCX]

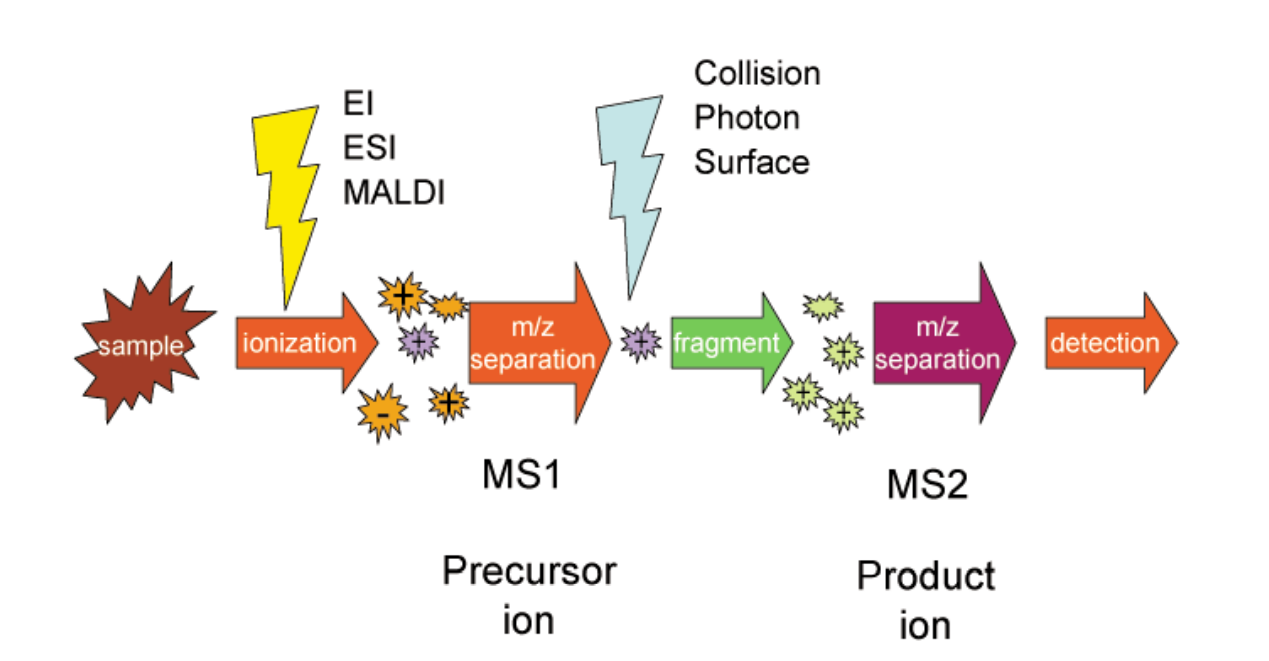


**Supplementary figure S1** Representing the methodology used for detection of different lipid compounds in the rind of six sugarcane genotypes.
